# Building a patient-centred nationwide integrated cardiac care registry: intermediate results from the Netherlands

**DOI:** 10.1007/s12471-024-01877-5

**Published:** 2024-05-22

**Authors:** Lineke Derks, Niki M Medendorp, Saskia Houterman, Victor A. W. M. Umans, Jos G. Maessen, Dennis van Veghel, all Registration Committees of the Netherlands Heart Registration

**Affiliations:** Netherlands Heart Registration, Utrecht, The Netherlands

**Keywords:** Public reporting, Quality data, Cardiology, Registries, Netherlands Heart Registration

## Abstract

This paper presents an overview of the development of an integrated patient-centred cardiac care registry spanning the initial 5 years (September 2017 to December 2022). The Netherlands Heart Registration facilitates registration committees in which mandated cardiologists and cardiothoracic surgeons structurally evaluate quality of care using real-world data. With consistent attendance rates exceeding 60%, a valuable network is supported. Over time, the completeness level of the registry has increased. Presently, four out of six quality registries show over 95% completeness in variables that are part of the quality policies of cardiology and cardiothoracic surgery societies. Notably, 93% of the centres voluntarily report outcomes related to open heart surgery and (trans)catheter interventions publicly. Moreover, outcomes after implantable cardioverter-defibrillator and pacemaker procedures are transparently reported by 26 centres. Multiple innovation projects have been initiated by the committees, signalling a shift from publishing outcomes transparently to collaborative efforts in sharing healthcare processes and investigating improvement initiatives. The next steps will focus on the entire pathway of cardiac care for a specific medical condition instead of focusing solely on the outcomes of the procedures. This redirection of focus to a comprehensive assessment of the patient pathway in cardiac care ultimately aims to optimise outcomes for all patients.

**Supplementary Information:**

The online version of this article (10.1007/s12471-024-01877-5) contains supplementary material, which is available to authorized users.

## Introduction

It is essential to monitor and improve quality of care, transparency, benchmarking and measurement of relevant quality indicators. Systematic documentation of real-world registry data is increasingly important as it includes all patient subgroups, enabling a structured evaluation of quality of care and additionally offering opportunities for research [1]. Worldwide, multiple clinical cardiac care quality registries exist that contain procedural and outcome data (e.g. SWEDEHEART [2], Society of Thoracic Surgeons Adult Cardiac Surgery Database (STS ACSD) [3], National Institute for Cardiovascular Outcomes Research (NICOR) [4] and the German Society for Thoracic and Cardiovascular Surgery (GSTCVS) registry [5]).

In the Netherlands, outcomes and quality of cardiovascular interventions and surgery are monitored within one national registry: the Netherlands Heart Registration (NHR). The NHR is a non-profit organisation that facilitates several nationwide, physician-driven quality registries covering all invasive cardiac interventional, electrophysiological and surgical procedures. The aim of the NHR is to use real-world data for patient-centred evaluation of quality of care. The NHR emerged in 2017 from collaboration between the Dutch societies for cardiologists and cardiothoracic surgeons (NVVC: Dutch Society for Cardiology; NVT: Dutch Association for Thoracic Surgery) and three separate quality registries [6]. This consequently enabled the creation of an integrated patient-centred cardiac care registry. Since its foundation, six intervention registries have been hosted: cardiothoracic surgery, percutaneous coronary intervention (PCI), transcatheter heart valve intervention (THI), ablation of atrial fibrillation (ablation), implantable cardioverter-defibrillator (ICD) implantation and explanation, and pacemaker.

Timely availability of accurate data, physician involvement, sufficient resources and the right culture are often mentioned as essential factors in registries or other programmes aiming to improve quality of care [7–12]. Within the NHR, the platform for learning and identifying improvement is primarily embedded in the established registration committees, in which mandated cardiologists and cardiothoracic surgeons represent their respective hospital. Inter alia, herein best practices in care delivery are identified and shared. More details on the NHR governance and processes have been thoroughly described elsewhere [6, 13].

**Fig. 1:**
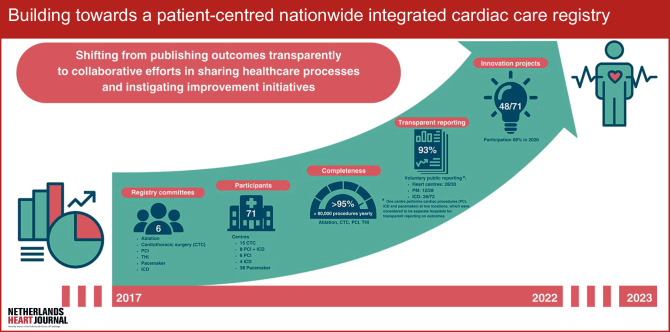
Infographic: Building towards a patient-centred nationwide integrated cardiac care registry. *CTC* cardiothoracic surgery, *PCI* percutaneous coronary intervention, *THI* transcatheter heart valve intervention, *ICD* implantable cardioverter-defibrillator

The aim of this article is to present intermediate results after 5 years of building an integrated patient-centred cardiac care registry. Results since its foundation are presented with respect to the primary processes of the NHR (Fig. 1).

## Methods

The NHR operates in the framework of three primary processes: registration, innovation and scientific research. These processes have been described extensively elsewhere [6] and are briefly summarised here. In this article, for each process several indicators are introduced and measured to assess development over time. In addition, client satisfaction was measured periodically and trends herein were evaluated. The period described in this paper covers September 2017 to December 2022.

### Registration

Registration focuses on the completeness and congruency of the data collected. In the Netherlands, there are 15 centres with cardiothoracic surgery onsite. One centre performs cardiac procedures (PCI, ICD and pacemaker) at two locations and was therefore, where appropriate, considered in the analysis to be two separate hospitals. Furthermore, there are 18 centres without cardiothoracic surgery onsite, of which 8 perform PCI and ICD procedures, and 6 and 4 centres solely perform PCI or ICD procedures, respectively. Finally, there are 38 centres which only perform pacemaker procedures. For each quality registry, cardiologists and/or cardiothoracic surgeons represent their centre on the registration committee; three cardiac anaesthetists represent national cardiac anaesthesiology involvement within the cardiothoracic surgery registration committee. The ICD and pacemaker registries are embedded in one registration committee, resulting in five registration committees. To evaluate attendance in each committee over time, a three-quarter running average over the period 2018–2022 was calculated.

Data from the predecessors were retained, and as the NHR became established, committees defined sets of variables for quality evaluation purposes. As part of the quality policies of the NVVC and NVT, it is mandatory for centres to register and submit certain variables to the NHR. The deadline for the centres to submit data from the preceding calendar year is 1 May. To assist hospitals in accomplishing high levels of data quality, the NHR has a data quality assurance system in place which is a requirement of the NEN-7510 certificate held by the NHR. These processes, including auditing to accomplish validated data within the NHR database, have been outlined in detail elsewhere [6, 13]. For registries covering interventions under the Special Medical Procedures Act (Dutch: Wet bijzondere medische verrichtingen (Wbmv)) completeness rates of 98% and above are targeted, while 95% completeness is the aim for the pacemaker registry. To evaluate completeness of registered mandatory variable sets, the number of valid elements was divided by the total number of mandatory elements per year. Completeness was additionally calculated for each centre separately to determine the number of centres meeting the intended rates.

### Innovation

Innovation involves new, progressive initiatives with the overarching aim of gaining further insights into, and potentially improving, quality of care. An innovation board for both value-based health care and artificial intelligence provides support and advice on projects within this pillar. One of the initiatives within ‘innovation’ is a voluntary public reporting programme [14]. In this programme, patient-relevant outcomes per centre for cardiac and cardiothoracic procedures are transparently and publicly reported, for example, to set benchmarks. The programme originally comprised outcomes after cardiothoracic surgery, PCIs, THIs and ablations. In 2019, this programme was expanded to reporting outcomes after ICD and pacemaker implantations or replacements.

Additionally, projects beyond the scope of the existing registries are initiated. This involves collecting additional data on specific quality concerns, like in-depth analyses of subgroups of patients. Moreover, projects may involve the development of new registries for quality purposes, such as patients diagnosed with heart failure who do not necessarily undergo a cardiac intervention. Innovation also aims to initiate projects focused on reducing registration burden while preserving centres’ resources without losing or even enriching quality information.

Centres’ participation in innovation projects is voluntarily and participation can be used as an indicator for project relevance and impact [15]. The extent to which registration committees initiate projects and the participation rate in innovation projects was therefore evaluated. In this article, several projects are summarised for further clarification in which we sought to present a picture of the diversity of the projects within all of the registration committees.

### Scientific research

The NHR enables researchers to re-use registry data to answer research questions conducive to improving quality of care. Requests are evaluated by the Scientific Council [16] and respective registration committees [6]. Evaluation is performed in accordance with specific criteria, i.e. (scientific) relevance of the research question, methodology, statistical analyses, privacy and potential risk of traceability to individual patients or hospitals. Data from multiple registrations can be requested for selection of specific subgroups of patients. For details on data requests, visit: https://nhr.nl/wetenschappelijk-onderzoek/. All granted data requests are made publicly available [17]. Data are made available in an aggregated form. Data that can be traced back to hospitals and/or patients are only made available with the explicit permission of the hospitals and/or patients involved. The Scientific Council monitors progression to the publication of results, which should preferably be within 1 year after acceptance of the request. The total number of accepted data requests per registry per year was counted. Research published using data from NHR quality registries or projects can be accessed online [18].

### Client satisfaction

Since 2018, the NHR’s image has been assessed annually among several stakeholders using a questionnaire (available from the corresponding author on request). Respondents evaluated performance with respect to several domains of data registration (11 aspects) and services (10 aspects) on a 5-point Likert scale. Finally, respondents answered the question ‘How likely are you to recommend the NHR to your colleagues?’ on a scale from 1 (strongly advise against) to 10 (strongly recommend). The Net Promoter Score (NPS) was calculated by subtracting the percentage of detractors (response score 1–6) from the percentage of promoters (response score 9 or higher). Results were also calculated separately for physicians versus other stakeholders.

## Results

### Registration

The NHR database currently covers over 1.5 million procedures. Yearly over 80,000 procedures are added for medical conditions such as coronary artery disease (approx. 40,000 PCIs, 6500 coronary artery bypass grafts), aortic valve disease (approx. 1200 surgical aortic valve replacements, 2500 transcatheter aortic valve implantations), and atrial fibrillation (approx. 5000 catheter ablations, 300 minimally invasive surgical ablations). Furthermore, approximately 12,000 pacemaker and 5800 ICD procedures are added yearly [19].

#### Registration committee attendance

**Fig. 2:**
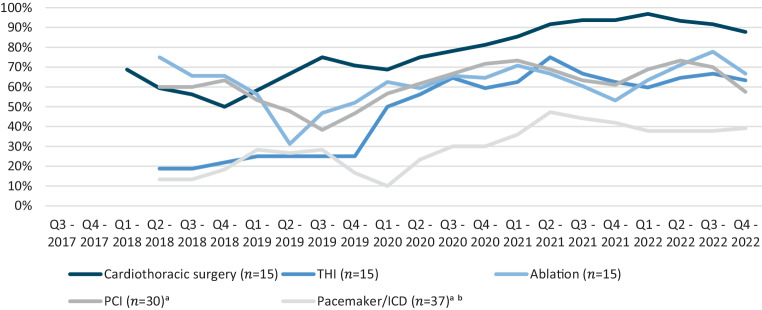
Attendance at registration committee meetings from 2018 to 2022 presented as a three-quarter running average with each line representing a different registration committee. *THI* transcatheter heart valve intervention, *PCI* percutaneous coronary intervention, *ICD* implantable cardioverter-defibrillator. ^a^One centre performs cardiac procedures at two locations, which are both represented on the registration committee as if they were two separate centres. ^b^A delegation of cardiologists performing pacemaker/*ICD* procedures represent their centre on the registration committee. Representatives of 30 centres up to 2021, thereafter 37

Figure 2 shows the attendance of mandated physicians at meetings as a percentage of the total number of centres that are represented on each registration committee. Attendance slightly decreased directly after the foundation of the NHR, but from mid-2019 onwards an increase in participation has been observed for all registries, most significantly for cardiothoracic surgery and THI. Nowadays, attendance is on average above 60%.

#### Completeness of data

**Fig. 3:**
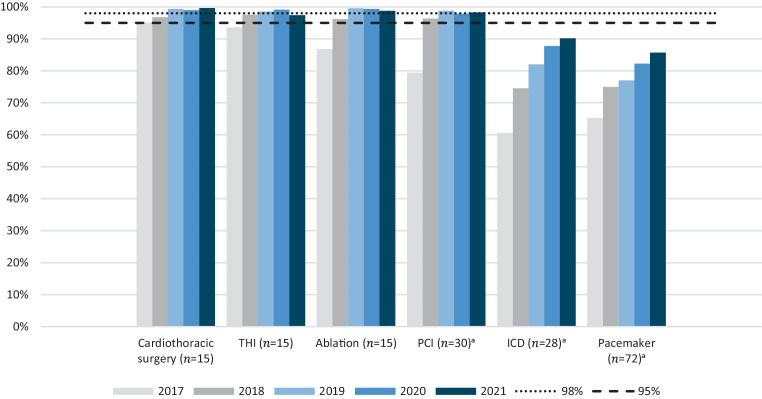
Completeness of the registration of the mandatory set of variables that are part of the quality policies of the Dutch Society for Cardiology and the Dutch Association for Thoracic Surgery for each registry separately (2017–2021). Completeness rates of 98% are targeted for interventions under the Special Medical Procedures Act (Dutch: Wet bijzondere medische verrichtingen (Wbmv)). The aim for the pacemaker registry is 95% completeness *THI* transcatheter heart valve intervention, *PCI *percutaneous coronary intervention, *ICD* implantable cardioverter-defibrillator. ^a^One centre performs cardiac procedures at two locations and is therefore considered in the analyses to be two separate centres

Figure 3 shows the completeness rate of mandatory variables for all separate registries over the past 5 years. The level of completeness has increased over time for all registries. Directly after the initiation of the NHR only between 3 (cardiothoracic surgery, ablation and PCI) and 7 centres (THI) met the completeness criteria, while for data from 2021 this rate increased to 13 of 15 centres for ablation, 13 of 15 for THI, 24 of 30 for PCI and 15 of 15 for cardiothoracic surgery.

### Innovation

#### Participation in voluntary public reporting programme

When the NHR was founded, 14 centres with cardiothoracic surgery onsite publicly reported on outcomes after cardiothoracic surgery, THI and ablation, and 21 centres (with and without cardiothoracic surgery onsite) reported on outcomes after PCI. A steady increase in participation has been observed over the years (Tab. 1). By 2022, almost all centres (28 out of 30) were voluntarily participating in the public reporting programme. Even though public reporting of outcomes after ICD and pacemaker procedures is lower (currently 12/28 and 26/72 centres, respectively), participation rates have rapidly increased since the start of the programme in 2018. The percentage of hospitals that participate, divided by all eligible hospitals per registry per year, is shown in Tab. 1.

**Table 1:** Number and percentage of centres participating in voluntary public reporting programme per registry per year, 2017–2022

Registry	2017	2018	2019	2020	2021	2022
Cardiothoracic surgery^a^, *n*/*N* (%)	14/16 (88)	14/16 (88)	14/16 (88)	14/16 (88)	14/15 (93)	14/15 (93)
THI^a^, *n*/*N* (%)	14/16 (88)	14/16 (88)	14/16 (88)	14/16 (88)	14/15 (93)	15/15 (100)
Ablation^a^, *n*/*N* (%)	14/16 (88)	14/16 (88)	14/16 (88)	14/16 (88)	14/15 (93)	15/15 (100)
PCI^b^, *n*/*N* (%)	21/30 (70)	21/30 (70)	22/30 (73)	24/30 (80)	26/30 (87)	28/30 (93)
ICD^b^, *n*/*N* (%)	*–*	*–*	5/28 (18)	6/28 (21)	7/28 (25)	12/28 (43)
Pacemaker^b^, *n*/*N* (%)	*–*	*–*	8/72 (11)	11/72 (15)	15/72 (21)	26/72 (36)

*THI* transcatheter heart valve intervention, *PCI* percutaneous coronary intervention, *ICD* implantable cardioverter-defibrillator

^a^In 2021 there was a decrease in the number of heart centres as a result of the fusion of two heart centres

^b^One heart centre performs cardiac procedures at two locations and is therefore considered in the analyses to be two separate centres

#### Innovation projects

During the first operational year, 39% (29/75) of all centres participated in at least one innovation project, and 50% (3/6) of the initiated projects involved five or more participating centres. This number has significantly increased to 67% of the centres (48/71) participating in one or more innovation projects. Currently, 13 of the 22 innovation projects (60%) involve five or more participating centres. All registry committees initiate innovation projects yearly, varying between one and four projects per registry.

Table S1 (Electronic Supplementary Material) shows an overview of the different types of innovation projects that have been initiated since the NHR has been operational, illustrated by concrete examples. As demonstrated, performing analyses on data of subgroups that are already available within the NHR, or collecting additional data on these groups for the purpose of addressing quality concerns, is initiated by all registration committees. The NHR simultaneously explores and builds on opportunities to gain information in addition to the current set of variables without increasing the registration burden.

### Scientific research

From its start until the end of 2022, the NHR received 96 data requests, of which 78 were accepted. There has been a steady increase from 4 requests in 2017 to 24 in 2021. Most accepted applications evaluate outcomes after cardiothoracic surgery (36/78, 46%). Furthermore, 22% (17/78) of the applications request data from two or more registries. An overview of data requests per registry per year is presented in Fig. S1 (Electronic Supplementary Material). Currently, 30 peer-reviewed papers, including position papers, registry data publications and NHR projects, have been published [18].

### Client satisfaction

**Fig. 4:**
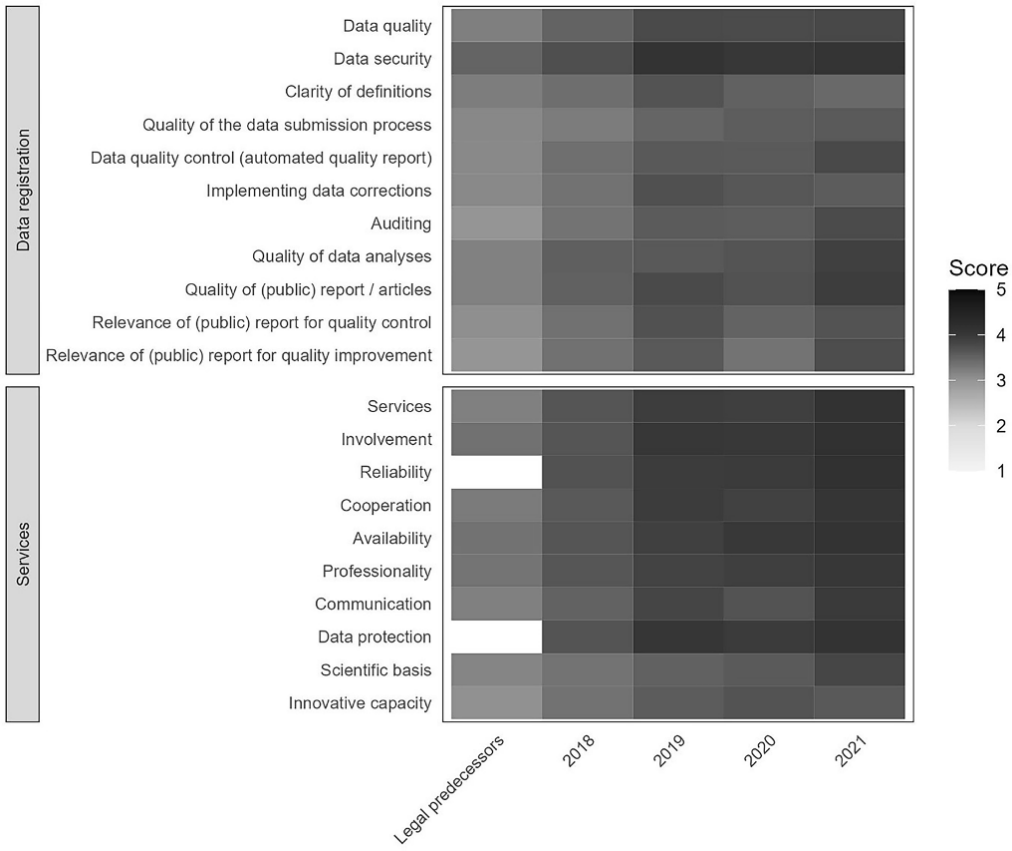
Average response on 5‑point Likert scale for aspects of data registration and services within the Netherlands Heart Registration per year (2018–2021) compared to legal predecessors

Summary results of client satisfaction per year are presented in Fig. 4. Compared to the legal predecessors the NHR scores higher across all aspects of data registration and services. On average, stakeholders have become increasingly more satisfied with the services of the NHR. Nowadays, 7 out of 10 aspects receive an average score of above 4 on the 5‑point Likert scale. In 2018, the NPS was negative (−6, *n* = 83), but the score gradually improved over time (2019 : 0, *n* = 50; 2020 : 10, *n* = 58) to a positive score of 25 (*n* = 52) in 2021. Compared to other stakeholders, physicians gave more positive feedback (Table S2 and Fig. S2, Electronic Supplementary Material).

## Discussion

In this article, we present intermediate results of the initial 5 years of building an integrated patient-centred cardiac care registry alongside the primary processes of the NHR. Although it is complex to establish its impact, several examples have shown demonstrable contributions of a cardiac care registry to quality of care [20, 21].

For most of the facilitated registries comprising over 1.5 million procedures, we showed high percentages of data completeness. The validity of the acquired data is ascertained by the embedded data quality assurance system and the audit process facilitated [13]. Extrapolating the positive trend, it is expected that in the coming years all NHR registries will attain the completeness goals of 95% or 98%. It should be noted, however, that adjustments to quality policies and related mandatory variables required by the NVVC and NVT may impact completeness rates, as centres need time to implement these changes within their electronic medical records in order to prospectively collect the data accordingly.

The mandated cardiologists and surgeons actively participate in registration committee meetings, contributing to improvement initiatives and the publication of scientific evaluations. Factors such as physician engagement and availability of complete and accurate data enable reliable benchmark analyses in the public reporting programme [7, 11, 12]. Public reporting may give rise to initiatives for improvement [9, 10]. In the past few years, we have observed in all registration committees a shift from publishing outcomes transparently to collaborative efforts in sharing healthcare processes with the aim of learning and generating ideas on further improving quality of care. This is supported by recent research by the NHR, showing that several centres actively use data and benchmark analyses to determine potential for improvement [22, 23]. In addition, results from several innovation projects initiated by the registration committee, addressing quality concerns such as differences in recovery from phrenic palsy, or gaining additional insights in patients with shock after PCI or in whom an extravascular device is implanted or replaced, have been published [24–26]. However, there is still a lot to be gained in this respect. Therefore, the NHR tries to support centres even further by organising meetings and providing a platform for exchanging content and approaches to projects.

In the past 5 years, the merging of cardiology and cardiothoracic surgery registries into the NHR has resulted in substantial progress towards one integrated patient-centred and nationwide cardiac care registry. The bundling of information on all cardiac interventions allows insight into index procedures and re-interventions by means of linking between the registries, enabling follow-up of cardiac patients while lowering the registration burden. Moreover, substantial steps towards a holistic perspective, i.e. the entire pathway of cardiac care, have been taken. The next step will include new registries focusing on medical conditions such as, but not limited to, heart failure, atrial fibrillation, endocarditis and congenital heart diseases. This enables the complete follow-up of patients, including those treated conservatively. The follow-up of these patients may provide relevant insights into guideline adherence and enable the determination of differences in outcomes between all groups of patients. Such insights may benefit physicians and their patients in shared decision-making and choosing wisely between treatment options.

Worldwide, there are several nationwide quality registries focusing on cardiovascular and cardiothoracic procedures, e.g. the GSTCVS registry, which is primarily based on voluntary unaudited survey data [5]. Other quality registries, such as the STS ACSD [27] and NICOR [4], have a multidiscplinary approach and focus on reliable national benchmarking and public reporting of patient-centred outcome measures. This aligns with the principles promoted by the non-profit International Consortium for Health Outcomes Measurement [28, 29]. Meanwhile, within the NHR, a physician-driven plan-do-check-act cycle has been established in the registration committees, initiating the necessary conditions to further build towards an integrated patient-centred cardiac care registry for disease-specific conditions. An example of a quality registry that focuses on specific medical conditions to evaluate the line of care involved is SWEDEHEART [2]. Its setup enables and successfully opens up the potential for registry-based randomised controlled trials (RBRCTs) [30, 31]. This allows SWEDEHEART to serve as an example for the ambitions of and steps taken so far by the NHR regarding the enabling of RBRCTs. Ultimately, clinical registries may become the platform for RBRCTs by utilising real-world data [32, 33].

In the Netherlands, several quality registration organisations exist that cover a variety of medical interventions and conditions. In various forms within these organisations, physicians have a central role in identifying and initiating improvement initiatives [34, 35]. However, the institution of registration committees as organised by the NHR, creating physician-driven registries, is considered unique. In this article we present medium to high participation rates of physicians at meetings of registration committees. The introduction of online meetings, in addition to vis-à-vis sessions, gained momentum due to the COVID-19 pandemic, stimulating and ensuring the continuity of the processes within the registration committee. Hence, a valuable network of involved physicians is continuously facilitated by the structure of the NHR. Physicians’ engagement and their satisfaction are correlated; when the policy and activities of the NHR are deemed relevant, reliable and of high-quality, the results are more likely to be actively discussed. The satisfaction of other stakeholders is additionally high and has shown a positive trend over time.

In conclusion, the NHR is the Dutch platform for registering validated quality data for cardiac procedures and is expanding to facilitate disease-specific quality registries. The data are essential in quality assessment for the NVVC and NVT, thereby contributing to clinical and governmental decision-making. The integration of complete and validated high-quality data, combined with active physician engagement, creates an environment where potential for improvement is identified, and new innovation projects are initiated, effectively supporting quality evaluation and improvement and creating the opportunity to move beyond evaluation of procedural quality to a comprehensive assessment of the patient pathway in cardiac care. Evaluating clinical data and processes should be dynamic and continuously updated and should therefore be one of the cornerstones of quality registries. Identification of essential outcomes is inevitable in updating registry variables and should therefore be published. Finally, the NHR enables re-use of registered data and is therefore suitable to support RBRCTs in the near future.

### Supplementary Information

Table S1 Examples of initiated innovation projects within the Netherlands Heart Registration between September 2017 and 2022

Table S2 Net Promotor Score (NPS) per year since 2018 by physicians and other stakeholders

Fig. S1 Number of accepted data requests applications per registry per year

Fig. S2 Average response on 5‑point Likert scale for aspects of data registration and services within the Netherlands Heart Registration by physicians (cardiologists and cardiothoracic surgeons) and other stakeholders for year 2021

List of members of the Registration Committees of the Netherlands Heart Registration
