# Supplementary material for: Building a patient-centred nationwide integrated cardiac care registry: intermediate results from the Netherlands
Source: Neth Heart J. 2024 May 22;32(6):228–37. doi: 10.1007/s12471-024-01877-5 (PMC11143093; doi:10.1007/s12471-024-01877-5)
Supplement: Supplementary file 1 — Table S1 Examples of initiated innovation projects within the Netherlands Heart Registration between September 2017 and 2022 [file 12471_2024_1877_MOESM1_ESM.docx]

**Table S1 Examples of initiated innovation projects within the Netherlands Heart Registration between September 2017 and 2022**

| Projects initiated within a registration committee  *Ablation:* Determine the best procedure strategy in patients with atrial fibrillation treated with pulmonary vein isolation.  *Ablation*: Analyses on difference in recovery from phrenic palsy between different methods of ablation after two years.  *Ablation*: Comparison of patients with same day discharge with patients with hospitalization longer than one day to examine whether differences in complications after procedure exist and tentative conclusions on safety of same day discharge can be drawn.  *Cardiothoracic surgery*: To be able to examine re-interventions after isolated aortic valve replacements, additional data on type and size of valve amongst other, is collected to gain insight into differences between different valves and whether it is beneficial to choose for a particular type of valve in some patient(group)s.  *Cardiothoracic surgery*: All patients who underwent a cardiothoracic procedure and died in the following period are analysed to identify whether they were at high risk prior to their procedure. The presumption is that high-risk patients act differently than low- and mediate-risk patients and including them in analyses with all patients may therefore be distortionary.  *THI*: Analysing hospitalization of different types of patients who underwent a transcatheter aortic valve implantation (TAVI) procedure to determine which patients has a longer hospitalization and for what reason.  *Pacemaker:* Collection of an additional set of variables for patients with pacemaker or ICD implantation in which at least one lead is removed that has been implanted more than one year ago or special tools are required to remove the lead(s) to gain insight into these specific procedures and patient groups.  *PCI:* Collecting data on patients who underwent a diagnostic procedure not necessarily followed by an intervention such as a PCI to gain insight into differences between hospitals and to determine if there is an association with outcomes.  *PCI*: Additional data of patients with cardiogenic shock prior to or after their PCI is collected and analysed to enable insight into this specific patient group. |
| --- |
| Projects concerning multiple registries  *Cardiothoracic surgery and THI*: Project in which additional data concerning the indication for surgical aortic valve replacement (SAVR) and TAVI is collected to examine whether patients < 75 years old who undergo SAVR differ from those who undergo TAVI.  *THI and PCI*: Analyses on TAVI patients that underwent a combined procedure with a PCI to identify whether these patients act differently than single procedure TAVI patients.  *Pacemaker and ICD*: The collection of data concerning a subpopulation of patients by whom a pacemaker or ICD is implanted, concerning patients by whom an extravascular device is implanted or replaced (eVDR project) or patients who receive antibiotics during their procedure and hospitalization.  *Ablation, Cardiothoracic surgery, PCI and THI (in collaboration with Harteraad, the patients federation)*: To determine how quality of life of patients undergoing a cardiac procedure can be better predicted beforehand, the NHR started a project in collaboration with some hospitals, in which additional data on potential determinants of quality of life is gathered among their patients. |
| Projects originally initiated by the NHR with the overarching aim to reduce registration burden (*always in collaboration with registration committees*)  *Facilitation of patients follow-up:* The NHR facilitates matching of registries within the NHR to enable patients’ follow-up, for example establishing whether patients who underwent TAVI or SAVR have had a pacemaker implementation during the period after their initial intervention.  *Collaboration between NHR and external organisations such as Dutch Hospital Data (DHD):* The NHR is searching for a close collaboration with external parties such as DHD to match data of patients present in both databases, to ensure hospitals do not need to register certain outcomes multiple times, such as hospitalization. |
| Projects involving the setting-up of new registries (*NHR in close collaboration with hospitals*)  In addition to the five intervention registries, the NHR has been working on new registries together with physicians from different hospitals. For example, registries involving patients with heart failure, atrial fibrillation, endocarditis and congenital heart diseases are in development in order to gain insight into these patients during their lifetime and answer research questions on these patient groups. |

*THI* transcatheter heart valve intervention, *ICD* implantable cardioverter-defibrillator, *PCI* percutaneous coronary intervention, *NHR* Netherlands Heart Registration
