# Supplementary material for: Building a patient-centred nationwide integrated cardiac care registry: intermediate results from the Netherlands
Source: Neth Heart J. 2024 May 22;32(6):228–37. doi: 10.1007/s12471-024-01877-5 (PMC11143093; doi:10.1007/s12471-024-01877-5)
Supplement: Supplementary file 2 — Table S2 Net Promotor Score (NPS) per year since 2018 by physicians and other stakeholders [file 12471_2024_1877_MOESM2_ESM.docx]

**Table S2 Net Promotor Score (NPS) per year since 2018 by physicians and other stakeholders.**

|  | **NPS 2018** | **N** | **NPS 2019** | **N** | **NPS 2020** | **N** | **NPS 2021** | **N** |
| --- | --- | --- | --- | --- | --- | --- | --- | --- |
| **Physicians (cardiologists and cardiothoracic surgeons)** | 0 | 47 | 37 | 19 | 0 | 24 | 32 | 19 |
| **Other stakeholders** | -11 | 38 | -26 | 36 | 18 | 34 | 21 | 33 |
| **Total** | -6 | 83 | 0 | 50 | 10 | 58 | 25 | 52 |
