# Supplementary material for: Building a patient-centred nationwide integrated cardiac care registry: intermediate results from the Netherlands
Source: Neth Heart J. 2024 May 22;32(6):228–37. doi: 10.1007/s12471-024-01877-5 (PMC11143093; doi:10.1007/s12471-024-01877-5)
Supplement: Supplementary file 3 — Fig. S1 Number of accepted data requests applications per registry per year [file 12471_2024_1877_MOESM3_ESM.docx]

**Fig. S1** Number of accepted data requests applications per registry per year
