# Supplementary material for: Building a patient-centred nationwide integrated cardiac care registry: intermediate results from the Netherlands
Source: Neth Heart J. 2024 May 22;32(6):228–37. doi: 10.1007/s12471-024-01877-5 (PMC11143093; doi:10.1007/s12471-024-01877-5)
Supplement: Supplementary file 4 — Fig. S2 Average response on 5‑point Likert scale for aspects of data registration and services within the Netherlands Heart Registration by physicians (cardiologists and cardiothoracic surgeons) and other stakeholders for year 2021 [file 12471_2024_1877_MOESM4_ESM.docx]

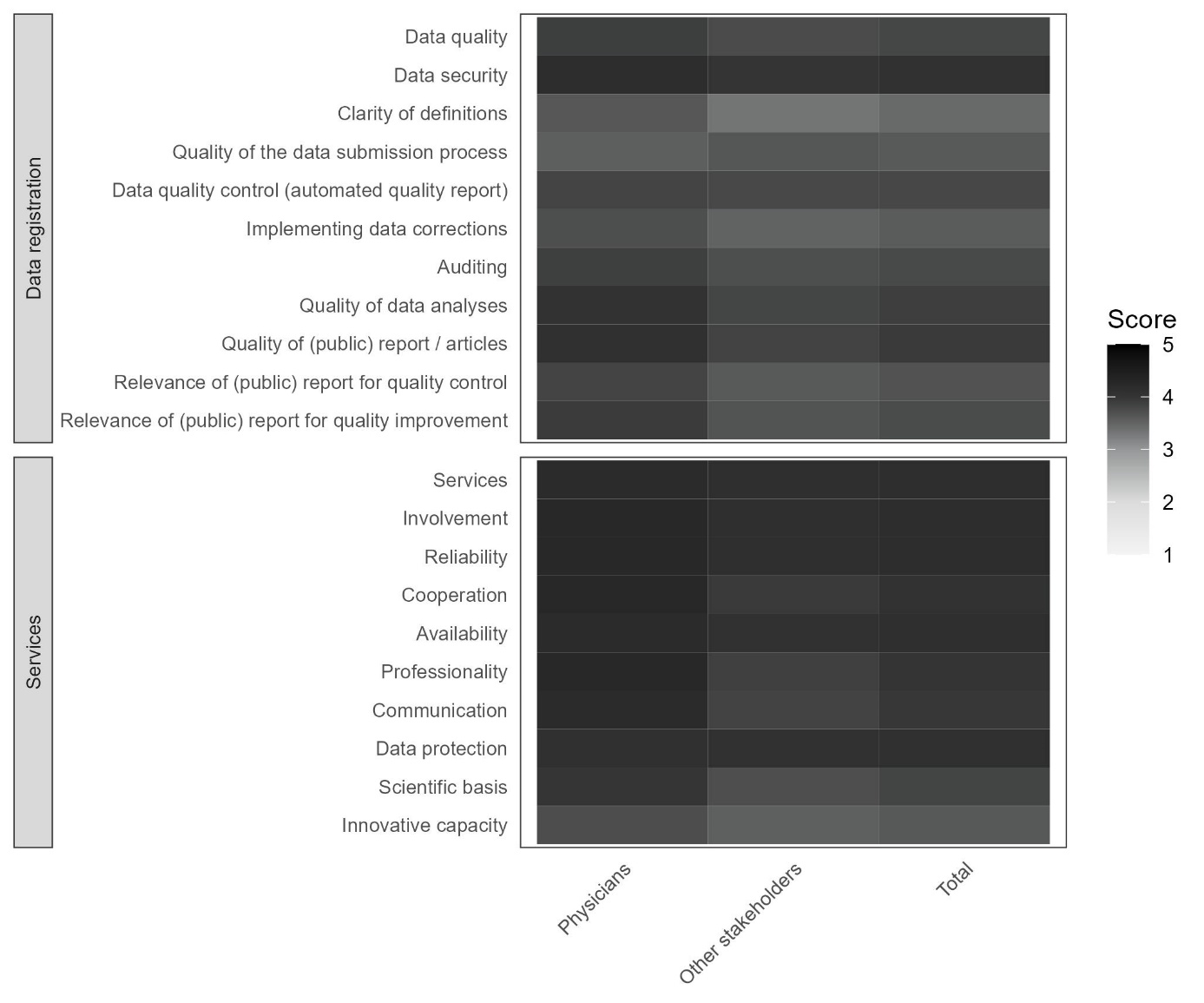
**Fig. S2** Average response on 5-point Likert scale for aspects of data registration and services within the Netherlands Heart Registration by physicians (cardiologists and cardiothoracic surgeons) and other stakeholders for year 2021
