# Supplementary material for: Building a patient-centred nationwide integrated cardiac care registry: intermediate results from the Netherlands
Source: Neth Heart J. 2024 May 22;32(6):228–37. doi: 10.1007/s12471-024-01877-5 (PMC11143093; doi:10.1007/s12471-024-01877-5)
Supplement: Supplementary file 5 — List of members of the Registration Committees of the Netherlands Heart Registration [file 12471_2024_1877_MOESM5_ESM.docx]

**LIST OF MEMBERS OF THE REGISTRATION COMMITTEES OF THE NETHERLANDS HEART REGISTRATION**

The following physicians are members of the Ablation Registration Committee of the NHR.

| W. | Kuijt | Amphia |
| --- | --- | --- |
| A.H.G. | Driessen | Amsterdam University Medical Centre, University of Amsterdam |
| M.J.B. | Kemme | Amsterdam University Medical Centre, VU Medical Centre |
| P | Van der Voort | Catharina Hospital |
| R.E. | Bhagwandien | Erasmus Medical Centre |
| J. | Van der Heijden | Haga Hospital |
| A | Adiyaman | Isala |
| S.A.I.P. | Trines | Leiden University Medical Centre |
| J.G.L.M. | Luermans | Maastricht University Medical Centre |
| K. | Kraaier | Medical Centre Leeuwarden |
| P.F.H.M. | Van Dessel | Medical Spectrum Twente |
| J.S.S.G. | De Jong | OLVG |
| S.W. | Westra | Radboud University Medical Centre |
| J.C. | Balt | St. Antonius Hospital |
| R.J. | Hassink | University Medical Centre of Utrecht |
| Y. | Blaauw | University Medical Centre Groningen |

The following physicians are members of the Cardiothoracic Surgery Registration Committee of the NHR.

| S | Bramer | Amphia |
| --- | --- | --- |
| R.A.F | De Lind van Wijngaarden | Amsterdam University Medical Centre, University of Amsterdam |
| A.B.A. | Vonk | Amsterdam University Medical Centre, VU Medical Centre |
| B.M.J.A. | Koene | Catharina Hospital |
| J.A. | Bekkers | Erasmus Medical Centre |
| G.J.F. | Hoohenkerk | Haga Hospital |
| A.L.P. | Markou | Isala |
| A. | De Weger | Leiden University Medical Centre |
| P. | Segers | Maastricht University Medical Centre |
| F. | Porta | Medical Centre Leeuwarden |
| R.G.H. | Speekenbrink | Medical Spectrum Twente |
| W. | Stooker | OLVG |
| W.W.L. | Li | Radboud University Medical Centre |
| E.J. | Daeter | St. Antonius Hospital |
| N.P. | Van der Kaaij | University Medical Centre of Utrecht |
| Y.L. | Douglas | University Medical Centre Groningen |

The following physicians are members of the Pacemaker/ICD Registration Committee of the NHR.

| C.J.H.J | Kirchhof | Alrijne Hospital |
| --- | --- | --- |
| W. | Kuijt | Amphia |
| J.R. | De Groot | Amsterdam University Medical Centre, University of Amsterdam |
| V.P. | Van Halm | Amsterdam University Medical Centre, VU Medical Centre |
| R.M.A. | Van de Wal | Bernhoven |
| I.R. | Henkens | Bravis Hospital |
| F. | Bracke | Catharina Hospital |
| J.J. | Wiersma | Dijklander Hospital |
| H.J. | Muntinga | Elisabeth-TweeSteden Hospital |
| R.E. | Bhagwandien | Erasmus Medical Centre |
| S.D.A. | Valk | Franciscus Gasthuis & Vlietland |
| J.W.L. | Hartog | Gelre Hospitals |
| R.W. | Grauss | Haaglanden Medical Centre |
| H. | Ramanna | Haga Hospital |
| J.J.H. | Bennik | IJsselland Hospital |
| A. | Adiyaman | Isala |
| R. | Joustra | Jeroen Bosch Hospital |
| F.M. | Horters | Maasstad Hospital |
| J.G.L.M. | Luermans | Maastricht University Medical Centre |
| M. | Smit | Martini Hospital |
| T. | Vromen | Máxima Medical Centre |
| E.A. | De Vrey | Meander Medical Centre |
| A.E. | Borger-van der burg | Medical Centre Leeuwarden |
| J.M. | Van Opstal | Medical Spectrum Twente |
| G.P. | Kimman | Noordwest Hospital Group |
| I.E. | Hof | OLVG |
| E.P. | Meindersma | Radboud University Medical Centre |
| F. | Brouwers | Rijnstate |
| A.B. | Van Zoelen | Rivas Hospital Group |
| I.C.D. | Westendorp | Rode Kruis Hospital |
| B.M. | Van Bemmel | Saxenburgh Medical Centre |
| V.F. | Van Dijk | St. Antonius Hospital |
| W.P.J. | Jansen | Tergooi |
| A.E. | Tuinenburg | University Medical Centre of Utrecht |
| A.H. | Maass | University Medical Centre Groningen |
| R. | Hazeleger | VieCuri Medical Centre |
| M.W.Z. | Basalus | Hospital GroupTwente |
| B. | Broers | Zuyderland Medical Centre |

The following physicians are members of the PCI Registration Committee of the NHR.

| J. | Cheng | Albert Schweitzer Hospital |
| --- | --- | --- |
| M. | Meuwissen | Amphia |
| J.P. | Henriques | Amsterdam University Medical Centre, University of Amsterdam |
| K.M.J. | Marques | Amsterdam University Medical Centre, VU Medical Centre |
| T. | Teeuwen | Catharina Hospital |
| H. | Al Hashimi | Canisius Wilhelmina Hospital |
| M. | Magro | Elisabeth-TweeSteden Hospital |
| J. | Daemen | Erasmus Medical Centre |
| B.J. | Sorgdrager | Haaglanden Medical Centre |
| C.E. | Schotborgh | Haga Hospital |
| R. | Snijder | Isala |
| J. | Polad | Jeroen Bosch Hospital |
| R. | Scherptong | Leiden University Medical Centre |
| M. | Van der Ent | Maasstad Hospital |
| A.J.W. | Van ‘t Hof | Maastricht University Medical Centre |
| F. | Spano | Meander Medical Centre |
| J. | Brouwer | Medical Centre Leeuwarden |
| M.G. | Stoel | Medical Spectrum Twente |
| A. | Dedic | Noordwest Hospital Group |
| G. | Amoroso | OLVG |
| C. | Camaro | Radboud University Medical Centre |
| P.W. | Danse | Rijnstate |
| J.P. | Van Kuijk | St. Antonius Hospital |
| E.K. | Arkenbout | Tergooi |
| W.T. | Ruifrok | Treant Zorggroep, Scheper Hospital |
| A. | Kraaijeveld | University Medical Centre of Utrecht |
| E. | Lipsic | University Medical Centre Groningen |
| S. | Aydin | VieCuri Medical Centre |
| R. | Erdem | ZorgSaam Hospital |
| A.J.W. | Van ‘t Hof | Zuyderland Medical Centre |

The following physicians are members of the THI Registration Committee of the NHR.

| B.J.L. | Van den Branden | Amphia |
| --- | --- | --- |
| MV | Vis | Amsterdam University Medical Centre, University of Amsterdam |
| W.A.L. | Tonino | Catharina Hospital |
| NMDA | Van Mieghem | Erasmus Medical Centre |
| C.E. | Schotborgh | Haga Hospital |
| R.S. | Hermanides | Isala |
| F. | Van der Kley | Leiden University Medical Centre |
| P. | Vriesendorp | Maastricht University Medical Centre |
| F | Porta | Medical Centre Leeuwarden |
| M.G. | Stoel | Medical Spectrum Twente |
| G. | Amoroso | OLVG |
| M | Van Wely | Radboud University Medical Centre |
| L | Timmers | St. Antonius Hospital |
| M | Voskuil | University Medical Centre of Utrecht |
| H.W. | Van der Werf | University Medical Centre Groningen |
